# siRNAmod: A database of experimentally validated chemically modified siRNAs

**DOI:** 10.1038/srep20031

**Published:** 2016-01-28

**Authors:** Showkat Ahmad Dar, Anamika Thakur, Abid Qureshi, Manoj Kumar

**Affiliations:** 1 Bioinformatics Centre, Institute of Microbial Technology, Council of Scientific and Industrial Research, Sector 39A, Chandigarh-160036, India

## Abstract

Small interfering RNA (siRNA) technology has vast potential for functional genomics and development of therapeutics. However, it faces many obstacles predominantly instability of siRNAs due to nuclease digestion and subsequently biologically short half-life. Chemical modifications in siRNAs provide means to overcome these shortcomings and improve their stability and potency. Despite enormous utility bioinformatics resource of these chemically modified siRNAs (cm-siRNAs) is lacking. Therefore, we have developed **siRNAmod,** a specialized databank for chemically modified siRNAs. Currently, our repository contains a total of **4894** chemically modified-siRNA sequences, comprising **128** unique chemical modifications on different positions with various permutations and combinations. It incorporates important information on siRNA sequence, chemical modification, their number and respective position, structure, simplified molecular input line entry system canonical (SMILES), efficacy of modified siRNA, target gene, cell line, experimental methods, reference etc. It is developed and hosted using Linux Apache MySQL PHP (LAMP) software bundle. Standard user-friendly browse, search facility and analysis tools are also integrated. It would assist in understanding the effect of chemical modifications and further development of stable and efficacious siRNAs for research as well as therapeutics. siRNAmod is freely available at: http://crdd.osdd.net/servers/sirnamod.

RNA interference (RNAi) phenomenon was described by A. Fire and coworkers in *Caenorhabditis elegans*^1^. They injected double stranded RNA that potentially and specifically interfere with the endogenous gene at mRNA level leading to its silencing^1^. RNA silencing pathway utilizes dicer to process long double stranded RNA (dsRNA) to 19–21 nucleotide small interfering RNAs (siRNAs) with 2-nucleotide unphosphorylated 3′ overhangs^2^. RNA induced silencing complex (RISC) incorporates siRNA antisense strand, resulting in cleavage of cognate mRNA target^3^ as shown in Fig. 1.

siRNAs are explored extensively in gene silencing experiments and therapeutics development^4^. However, their therapeutic usage is hampered due to many factors e.g. susceptibility to ribonucleases digestion, short biological half-life, lack of proper delivery vehicle, cellular uptake, immune-stimulatory effect, non-specific gene targeting and toxicity^5^,^6^. These limitations are due to the inherent physicochemical properties of siRNAs that includes short and stiff structure, high charge density (poly-anionic), hydrophilic and liability to nuclease cleavage^5^,^6^. To mitigate these restraints, chemical modifications in siRNAs have been substantially investigated^7^.

Synthetic oligonucleotides were used in the late 1970’s for gene inhibition known as antisense technology^8^. Modified nucleoside phosphoramidites are used in solid phase chemical synthesis of siRNA which allow site-specific incorporation of chemically modified nucleotide moiety at specific positions within the siRNA^9^. Chemical modifications on siRNA are categorized based on component of nucleotide modified as ribose, base or phosphate and change in properties like biological activity, thermodynamic stability and nuclease resistance^7^. Chemical modifications on sugar moiety e.g. locked nucleic acids (LNA), unlocked nucleic acids (UNA)^10^,^11^, 2′-deoxy, 2′-O-methyl^12^, 2′-fluoro, 2′-methoxyethyl, 2′-aminoethyl, were tested for RNAi activity^13^,^14^. In LNA, methylene bridge is created between 2′-oxygen and 4′-carbon to increase the RNAi activity favorably^15^,^16^ and also improves nuclease resistance^17^. UNA are open ring derivatives of ribose without C-2′ and C-3′ carbon-carbon bond but structurally mimic the RNA when incorporated into duplex^18^. Ribose 2′-OH and phosphorothioate modification increases stability of siRNA^19^,^20^. Besides the 2′-OH was not found to be essential for RNAi activity^21^. However, fluorine modification at 2′-OH on different nucleotide positions of siRNA maintains silencing potency as well as increases thermal stability^22^,^23^. Further, researchers carried out various chemical modifications in different combinations and tested them for RNAi activity^13^,^14^. Also 2′-O-aminoethyl addition on passenger strand (sense strand) at 3′-end enhanced RNAi activity of siRNA duplex due to asymmetry in thermodynamic stability^24^.

Base modifications have been used in various studies e.g. hypoxanthine, 2,4-di-fluorotoluene, dihydrouridine, 2′-thiouridine, pseudouridine^12^,^25^,^26^ etc. Modification in this component can contribute towards better understanding of off-target effects as well as gene silencing mechanism^27^. Besides, phosphate modifications were also employed in different studies to enhance the properties of siRNAs^28^,^29^. These modifications include phosphorothioate, boranophosphate, peptide nucleic acids (PNA)^28^,^29^ etc. For instance phosphorothioate enhances the stability, uptake and biodistribition of the siRNA^30^,^31^. Large-scale screens and high throughput platforms using different permutation and combinations of chemical modifications were explored to check their effect^24^,^32^. Native (un-modified) siRNA show immunological activation *in vitro* to various degrees, while certain chemical modifications (2′-O-methyl) of siRNA avoid immunological effects^33^. Chemical modifications may be useful to overcome the obstacle of delivery of siRNAs without compromising their gene silencing activity^5^.

As discussed, next generation siRNA technology will be defined by the chemistry of modifications on siRNAs to greater extent. But we currently lack the platform to explore the complete spectrum of chemical modifications synthesized and plotted with relation to siRNAs so far. To describe the rules governing the performance of these cm-siRNAs, we need to have birds-eye view on the entire set of chemical modifications. Furthermore, positions of modifications on sense and antisense strands, modified nucleotide component, chemical features like size, functional groups, etc. need to be investigated thoroughly. Other associated experimental details like target gene, transfection method, cell line or organism used are very important and needs to be considered before further investigation. Additionally, the data needs to be present in the format that can be easily retrieved and may be utilized for further computational analysis.

Many repositories for native (unmodified) siRNA are available in the literature like siRecords^34^, siRNAdb^35^, human specific siRNA database (HuSiDa)^36^, viral siRNAs (VIRsiRNAdb)^37^ and HIV specific siRNA (HIVsirDB)^38^. Also for naturally occurring modified RNA, there is a database namely RNAMDB^39^. The latter provides comprehensive listing of the post transcriptionally modified nucleotides occurring naturally as in messenger RNA (mRNA), ribosomal RNA (rRNA) and transfer RNA (tRNA). But no dedicated repository has been developed yet for cm-siRNA despite their usefulness. Therefore, we developed siRNAmod that will serve as the central repository of the experimental records of chemical modification of siRNAs.

## Results

### Database statistics

**Figure 2:**
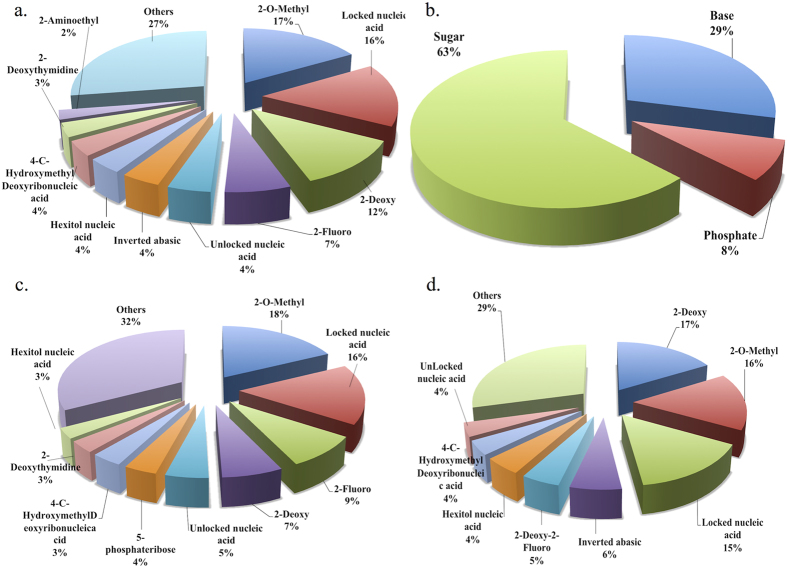
Chemical modification statistics pie charts (**a**) Overall siRNA modifications, (**b**) nucleotide component based (sugar, base phosphate), (**c**) Antisense strand modification distribution and (**d**) Sense strand modification distribution.

siRNAmod contains 4894 experimentally validated cm-siRNA entries. 128 unique chemical modifications incorporated on various components of nucleotides are archived. The overall statistics of the chemical modifications is depicted in Fig. 2. Chemical modifications distribution is illustrated in Fig. 2a. Majority of the reported modifications executed are 2′-O-methyl, locked nucleic acid, 2′-deoxy, 2′-fluoro and unlocked nucleic acid with 17%, 16%, 12%, 7%, and 4% respectively. Both 2′-O-methyl and 2′-deoxy are natural RNA modifications. The percentage distribution of natural and non-natural chemical modifications of cm-siRNAs is shown in supplementary Fig. S1. The individual distribution of chemical modifications for antisense and sense siRNA strands is represented in Fig. 2c,d correspondingly. Main modifications are directed on ribose with 63% followed by base and phosphate modifications with 29% and 8% respectively (Fig. 2b). The chemical modifications of the base moiety include hypoxanthine, 2,4-difluorotoluene, boron cluster. While phosphate modifications contain phosphorothioate, boranophosphate, peptide nucleic acid, etc.

**Figure 3:**
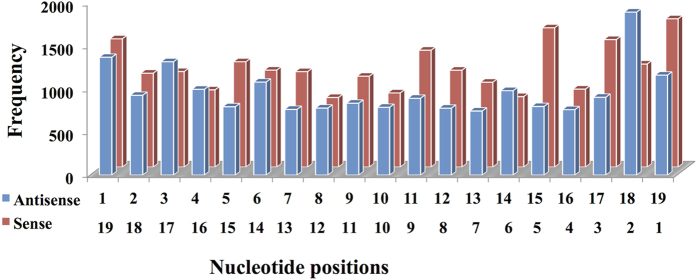
Reverse complement based chemical modification of siRNA position wise on sense and antisense siRNA sequences.

Complementarity based sense and antisense distribution of chemical modifications is shown in Fig. 3. Statistical trend of cumulative distribution and combinations of different chemical modifications is represented in supplementary Fig. S3 and S4. Different experimental methods like luciferase assay, RT-PCR, northern blotting etc. were used to validate cm-siRNAs. Most common method to evaluate the efficacy was luciferase assay. The cm-siRNA stability was checked using melting temperature experiments or thermodynamic stability. Transfection methods used on different cell lines mainly utilized lipid based reagents viz lipofectamine and Interfer (Polypus-Transfection). Percentage efficacy ranges from 0–100. However, negative efficacy values were also reported with respect to control taken in a particular experiment. Therefore, for the sake of simplicity, we have reported negative efficacies as zero.

For experimentation, both *in vivo* and *in vitro* systems were reported in the literature. Commonly used cell lines were HeLa, HEK 293, Hepa 1–6, Vero, SH-SY5Y etc. Further, *in vivo* system includes Balb C mice, C57BL/6 mice etc. Pie chart depicting their distribution is presented in supplementary Fig. S5. 128 unique chemical modification structures used to modify the siRNA nucleotides are organized in the form of list with hyperlinks to information of individual modification. Here, we have combined the similar types of modifications into single categories as synonyms to represent the same or similar modifications reported in different studies.

### Database browsing

siRNAmod browsing option helps user to surf via these five categories: i) modification in sense strand, ii) position of modification in sense strand, iii) modification in antisense strand, iv) position of modification in antisense strand and v) reference. Modifications that include more than one chemical moiety on same strand are separated by the asterisk (*) symbol. “0” modification in the fields of sense or antisense strand indicates that the modification is present on the other strand.

Simultaneously, facility to navigate 128 chemical modifications is also provided. This offers user an overview of chemical moieties used for modification to modulate siRNA properties. Clicking on the individual modification, structure and related chemical information about the chemical moiety is displayed via jmol^40^.

### Database search

**Figure 4:**
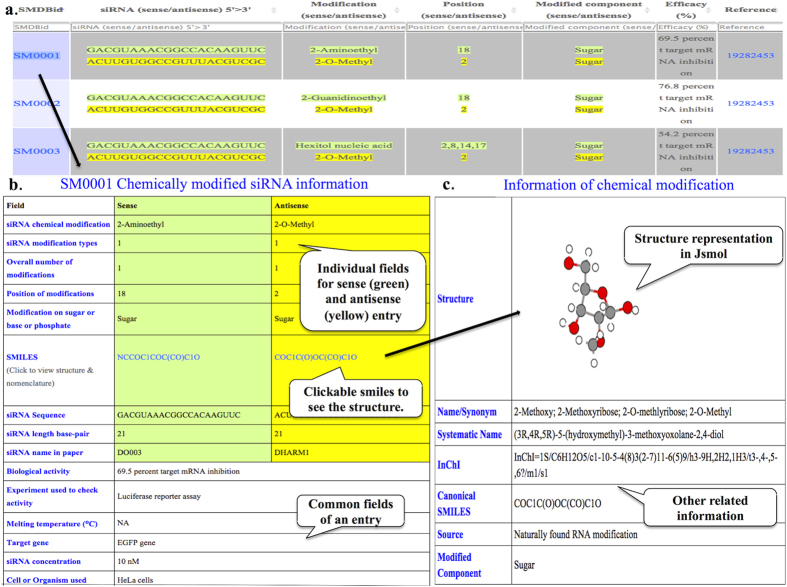
Display of search output with cm-siRNA details (**a**) Result output of search or browse (**b**) Details page of individual entry (**c**) Structure and other chemical information of modification.

There are two search options provided (i) basic search (ii) structure based search. For basic search, user can enter keyword in query-box and find relevant information. User can choose exact or containing operators. As indicated from the name, “*exact”* option is the strict search mode, which finds the exact term that user provides while *“containing*” is lenient search option and provides wider results. The output is displayed in Fig. 4a. On clicking individual Id, user can look for its detail. Further, structure of the chemical modification can be viewed by clicking on the SMILES link as provided (Fig. 4b,c). The result output can be filtered and sorted by clicking or writing on the column headers.

For structure drawing and search, MarvinSketch tool is provided. Chemical structures can be hand drawn and used to search for their presence or absence in the database. Step by step instructions for searching database and its output are displayed using screen shots in “How to use” menu on the web server.

### Tools

*siRNAmod-BLAST* provides similarity search option to look for siRNA sequences against the database. This will help the user to know whether similar siRNA sequences are already reported in database or not. *siRNAmod-Map* enables the user to map cm-siRNAs reported in the database against a particular nucleotide sequence. In addition, we have also provided submit option for new chemical entries by various researchers. Further help page and other related links are provided.

### Implementation

Database front end is developed using Perl, PHP, JavaScript HTML and CSS. It is maintained on MySQL server using LAMP software solution. The resource is hosted on IBM SAS ×3800 machine. siRNAmod server and its integrated tools are compatible with all browsers like Google Chrome, Mozilla Firefox and Safari. However, structure based search tool (MarvinSketch) needs java to be enabled on the browser.

## Discussion

siRNAs are being actively tested as new potential therapeutics against various disorders as well as pathogens^37^,^41^,^42^. More than 20 siRNA based therapeutics are currently at different phases of clinical trials^43^. Examples of such cm-siRNA drugs are SPC2996, EZN3042 and SPC3649 for leukemia, solid tumors, and Hepatitis C virus (HCV) infection respectively^43^. Chemical modifications remain the critical aspect for development of siRNA therapeutics to reduce their inherent limitations^6^,^44^. For example, 2′-fluoro, 2′-O-methyl, 2′-deoxy, unlocked nucleic acid, 2′-hydroxy and phosphorothioate modification increase siRNA serum stability^19^,^20^. Likewise, certain nucleotide motifs in siRNA sequence as UA and CA upon modification with 2′-O-methyl or 2′-fluoro enhances the nuclease resistance^45^. Locked nucleic acid, 2′-O-methyl, 2′-fluoro moieties thermodynamically stabilize siRNA ends while unlocked nucleic acid, phosphorothioate, ethylamino, dihydrouracil modifications destabilize them^46^. Stabilizing the 5′-end and destabilizing the 3′-end of passenger strand enhances the potency of siRNA and vice versa^46^. siRNAs of length 19 to 21 base pairs are less immunogenic than longer ones^47^. Modifications like 2′-thiouridine, 4′-thiouridine, 2′-deoxyuridine reduce Protein kinase R (PKR) activation^48^ while 2′-fluoro, 2′-deoxy, and 2′-O-methyl modifications abolish Toll like receptor (TLR) interaction preserving silencing activity even upon heavy modification^46^.

Various comprehensive studies in this field have been conducted which employed new strategies and methods. Bramsen and coworkers^24^ utilize 21 different chemical modifications on various positions of sense and antisense strands. Both strands were then combined to generate permutations and combinations of cm-siRNAs. Butora *et al.* employed walkthrough method^13^,^32^, which replaces single or multiple nucleotides in siRNA with different modifications sequentially from one end to another and checking their biological impact. Further effect on enhanced efficacy due to chemical modifications was tested using different siRNAs concentrations^49^. Further, compatibility of RNAi protein machinery with cm-siRNA format has been investigated^50^,^51^. Peel *et al.* examined target degradation efficacy of small molecules conjugated to siRNAs^52^. Similarly, other groups also studied terminal modifications like 5′-phosphate^53^ or serinol modification^54^.

We plotted overall database chemical modifications statistics. Complementary positions from 1 to 19 of cm-siRNAs show comparatively similar frequency distribution of cumulative modifications as depicted in Fig. 3. Overall statistical trend of chemical modifications depicts that terminal positions 1, 2, 3 and 20, 21 are highly altered comparative to central portion as shown in supplementary Fig. S2. This may be probably due to ease of end modifications and their role in nuclease resistance^55^. Further, antisense seems a bit less modified than sense strand, may be due to their differential roles in RISC-machinery^56^. Hence, modifying each strand distinctly may prove a handy tool to improve their cm-siRNA functioning.

Pattern of cumulative occurrence of nucleotides modified on sense and antisense strand of cm-siRNAs is depicted in supplementary Fig. S3. It reveals that majorly one, two or three nucleotides have been engineered for chemical modifications on either siRNA strand. However only in limited studies nucleotides on all twenty-one positions in a siRNA have been modified. Reason for this could be the toxicity associated with increased modifications^57^. Combinations of different unique modifications i.e. how many exclusively different chemical moieties are integrated in cm-siRNA is displayed in supplementary Fig. S4. It elucidates that maximum 5 different combinations of modifications are seen so far in cm-siRNAs. These conglomerates need to be optimized for construction of biologically significant siRNAs^24^. So, there is further scope to explore these combinations.

For data retrieval and usage we have designed user-friendly tools and web pages. Users can select the unique chemical modification list on web server and check their patterns i.e. on what positions the modifications are reported along with their biological effects. We have provided the browse option with respect to modifications and positions on either sense or antisense strand of siRNA. User can also analyze cm-siRNA sequences based on the positions of modifications. Furthermore, structure search option is also available to draw the structure and search against the database. The database would be updated half yearly/annually or on availability of the enough data in the literature.

## Methods

### Data acquisition

Literature was exhaustively searched for experimentally validated chemically modified-siRNAs (cm-siRNAs) for extraction of relevant information using keywords related to (i) RNAi (ii) modifications (iii) stability, utilizing advanced search option in PubMed. The search resulted in about 900 articles as on June 2015, which we examined for relevant experimental data on cm-siRNAs. Studies related to chemical modification for their synthesis, lacking complete biological information and reviews were excluded. About 500 potential articles were scrutinized to mine the required fields. Finally from 96 articles, comprehensive information of 4894 cm-siRNAs was extracted.

We have also integrated chemical information about modifications introduced in the siRNAs. This information is derived form public chemical databases namely PubChem^58^, ChemSpider (http://www.chemspider.com), ChEMBL (https://www.ebi.ac.uk/chembl). Structures of some chemical modifications were hand drawn using MarvinSketch package (http://www.chemaxon.com), as they were not available in above chemical repositories.

### Database organization

**Figure 5:**
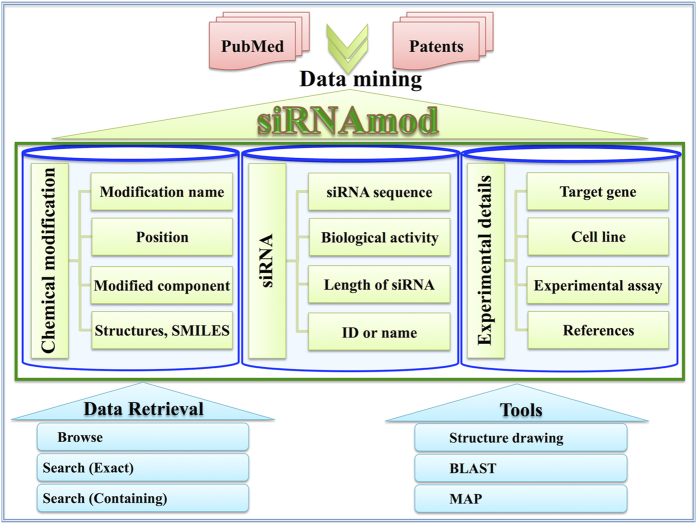
Diagrammatic representation of siRNAmod architecture.

siRNAmod provides comprehensive information on experimentally validated cm-siRNA sequences and their chemical modifications. Database is structured to provide information in more than 20 fields broadly divided into; (A) Chemical modification (B) siRNA sequence (C) siRNA activity (D) Experimental details. The database architecture is shown in Fig. 5.

#### (A)Chemical modification

It includes the information about the chemical moiety and all its related details for both sense and antisense strand distinctly.

(i) Name of chemical modifications for example “2-O-methyl”. If multiple modifications are present then they are separated by asterisk (*) mark e.g. “ 2,4-Bridged nucleic acid* Deoxythymidine”; (ii) their unique and cumulative numerical magnitude (iii) positions of chemical modifications i.e. positions on the siRNA sequence where the modifications are incorporated. When one modification is present at more than one position, it is represented by the position separated by comma e.g. “1,2,3,4,5” and two modifications at respective positions are separated by asterisks as “1,2,3,4,5 * 23,24”; (iv) modification component of nucleotide presents the information about the modified substructure of the nucleotide i.e. whether sugar, base or phosphate part is modified; (v) structures of modification; (vi) SMILES (simplified molecular input line entry system canonical), InCheI (International Chemical Identifier); (vii) systematic name etc.; (viii) natural or non-natural presence of chemical modification.

#### (B) siRNA sequences

It encompasses information relevant to cm-siRNA sequences and includes (ix) sequence of unmodified siRNA i.e. the basic sequence on which the modification is performed both sense and antisense; (x) length of the siRNA; (xi) Id or name of siRNA used in the respective article. Besides, 435 unmodified siRNAs that were used as controls in the respective studies were also extracted and provided separately in the database.

#### (C) siRNA activity

It comprises of (xii) biological activity or efficacy of the cm-siRNA as percentage of target mRNA degraded. Besides, inhibition concentration-50 (IC_50_) values are sometimes provided in the studies instead of percentage inhibition. Qualitative representation of activity is also used to represent the activity of modified siRNA where exact quantitative values were not given, using the terms like high or low activity comparatively.

#### (D) Experimental details

It covers rest of the relevant information as (xiii) target gene; (xiv) melting temperature of modified siRNA sequence; (xv) siRNA concentration; (xvi) experiment used to check activity; (xvii) cell line or organism; (xviii) transfection method; (xix) post transfection duration; (xx) reference.

## Conclusion and Future Developments

Chemical modifications in siRNAs are one of the inevitable steps to ameliorate their therapeutic potential. siRNAmod is the first comprehensive platform of 4894 cm-siRNAs with 128 unique chemical modifications. It provides manually curated information about chemical modification, sequences, siRNA activity and experimental details. Chemical information includes structure, SMILES, InCheI to make the database chemically aware. User-friendly web interface is provided to retrieve the desired curated information with ease. Exploration of chemical modifications and analysis in siRNAs and has been accelerated in last decade and data is continuously growing. In future, we would update the database and include cm-siRNA based design tools and algorithm. We hope that siRNAmod would be helpful for further analysis, interpretation and to accelerate the development of siRNA-based therapeutics.

## Additional Information

**How to cite this article**: Dar, S. A. *et al.* siRNAmod: A database of experimentally validated chemically modified siRNAs. *Sci. Rep.* **6**, 20031; doi: 10.1038/srep20031 (2016).

## Supplementary Material

Supplementary Information

## Figures and Tables

**Figure 1:**
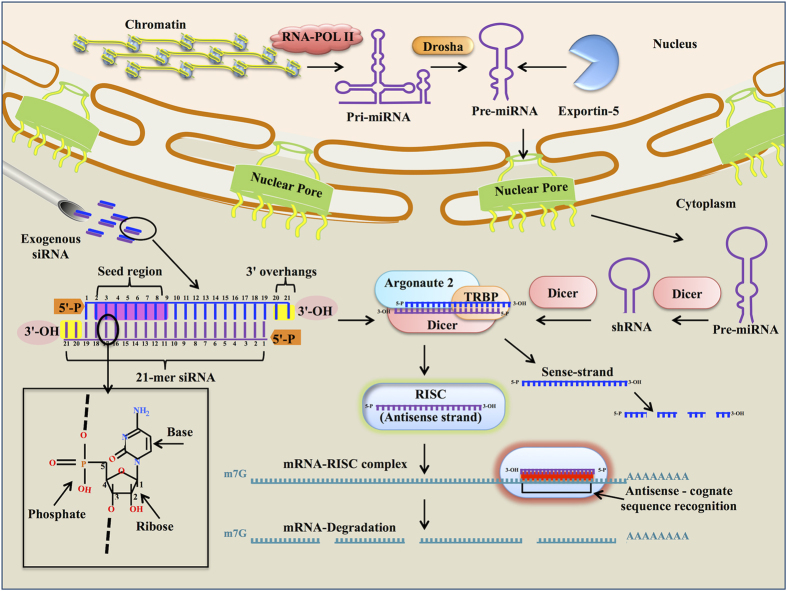
Cartoon representation of RNAi mechanism, siRNA molecular structure and its entry in RNAi pathway.
