# Supplementary material for: siRNAmod: A database of experimentally validated chemically modified siRNAs
Source: Sci Rep. 2016 Jan 28;6:20031. doi: 10.1038/srep20031 (PMC4730238; doi:10.1038/srep20031)
Supplement: Supplementary Information [file srep20031-s1.pdf]

siRNAmod: A database of experimentally validated chemically modified siRNAs

Showkat Ahmad Dar[1], Anamika Thakur[1], Abid Qureshi[1] and Manoj Kumar[1,*]

1 Bioinformatics Centre, Institute of Microbial Technology, Council of Scientific and Industrial Research, Sector 39A, Chandigarh-160036, India

* To whom correspondence should be addressed: Tel.: +91-172-6665453; Fax: +91-172-2690585, 2690632; Email: manojk@imtech.res.in

Showkat Ahmad Dar
Bioinformatics Centre, Institute of Microbial Technology, Council of Scientific and Industrial Research, Sector 39A, Chandigarh-160036, India
showkat@imtech.res.in

Anamika Thakur
Bioinformatics Centre, Institute of Microbial Technology, Council of Scientific and Industrial Research, Sector 39A, Chandigarh-160036, India
anamikathakur@imtech.res.in

Abid Qureshi
Bioinformatics Centre, Institute of Microbial Technology, Council of Scientific and Industrial Research, Sector 39A, Chandigarh-160036, India
qureshisaab@imtech.res.in

Manoj Kumar
Bioinformatics Centre, Institute of Microbial Technology, Council of Scientific and Industrial Research, Sector 39A, Chandigarh-160036, India
manojk@imtech.res.in

Additional files

Additional file 1

Figure S1. Distribution of natural and non-natural chemical modifications.

Figure S2. Relative frequency of chemical modifications on sense and antisense cm-siRNAs for 21 base pair.

Figure S3. Cumulative frequency of cm-siRNA modifications on sense and antisense sequences.

Figure S4. Grouping frequency of different unique chemical modifications.

Figure S5. Cell lines distribution in siRNAmod database.

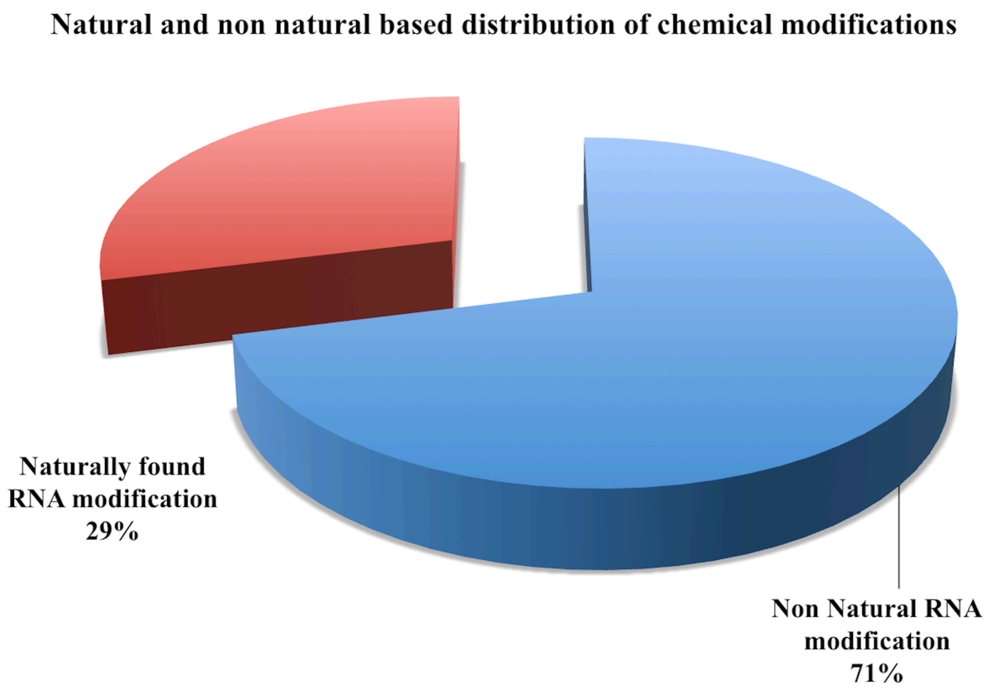

Figure S1. Distribution of natural and non-natural chemical modifications.

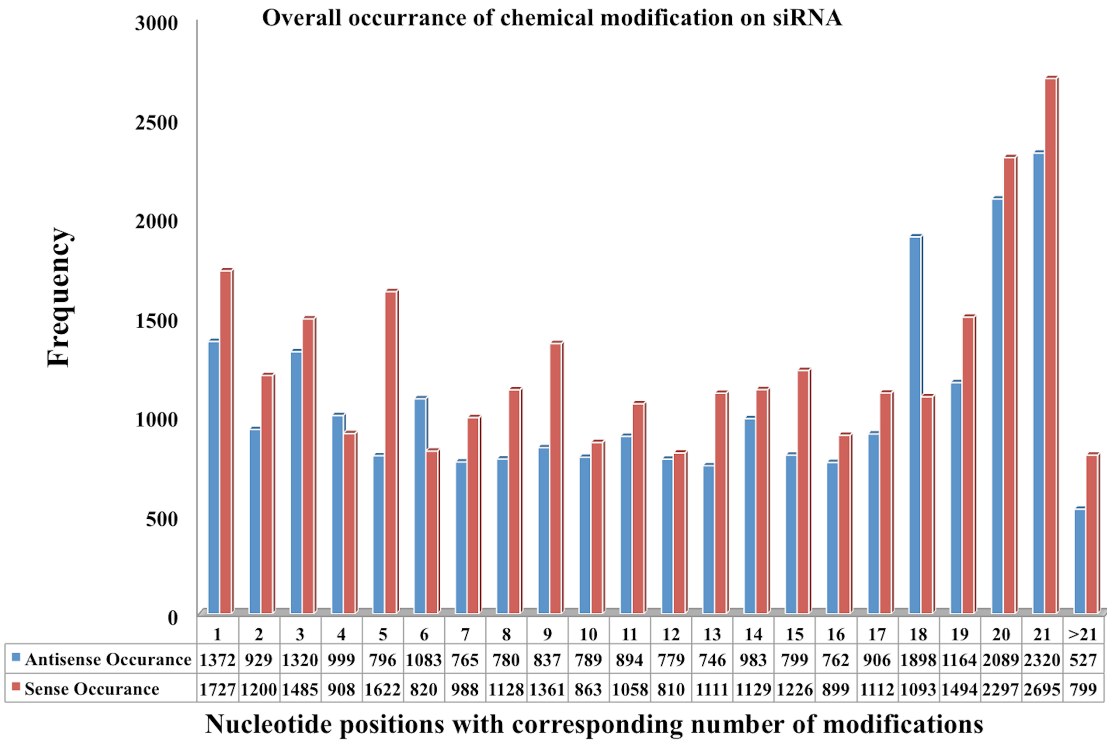

	1	2	3	4	5	6	7	8	9	10	11	12	13	14	15	16	17	18	19	20	21	>21
Antisense Occurance	1372	929	1320	999	796	1083	765	780	837	789	894	779	746	983	799	762	906	1898	1164	2089	2320	527
Sense Occurance	1727	1200	1485	908	1622	820	988	1128	1361	863	1058	810	1111	1129	1226	899	1112	1093	1494	2297	2695	799

Nucleotide positions with corresponding number of modifications

Figure S2. Relative frequency of chemical modifications on sense and antisense cm-siRNAs for 21 base pair. Nucleotide position represented as 1 to 21 both starting from 5' end on X-axis. >21 signifies end modifications or additional modifications attached after 21st nucleotide. Further number of each position is provided below its corresponding position.

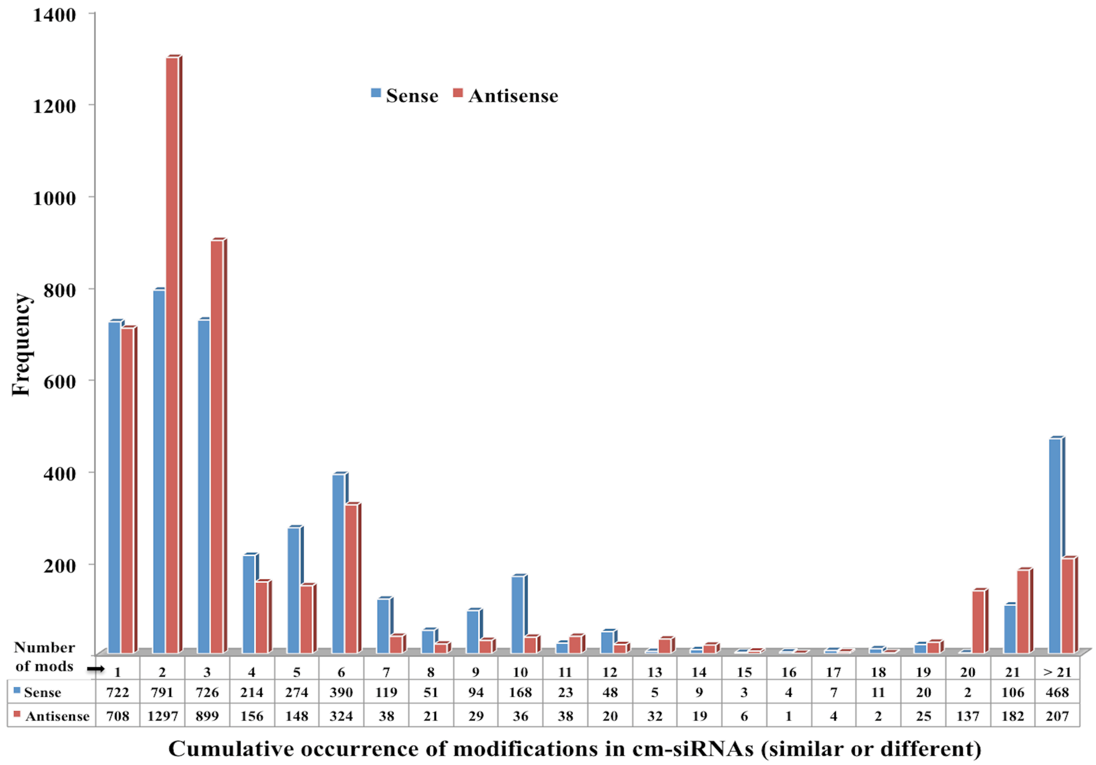

Number of mods →	1	2	3	4	5	6	7	8	9	10	11	12	13	14	15	16	17	18	19	20	21	> 21
Sense	722	791	726	214	274	390	119	51	94	168	23	48	5	9	3	4	7	11	20	2	106	468
Antisense	708	1297	899	156	148	324	38	21	29	36	38	20	32	19	6	1	4	2	25	137	182	207

Cumulative occurrence of modifications in cm-siRNAs (similar or different)

Figure S3. Cumulative frequency of cm-siRNA modifications on sense and antisense sequences. Number of Mods on X-axis represents the cumulative number of modifications on cm-siRNAs i.e. total number of modifications (same or different) are present on the particular cm-siRNAs. Their respective numbers are also provided in proceeding rows on X-axis.

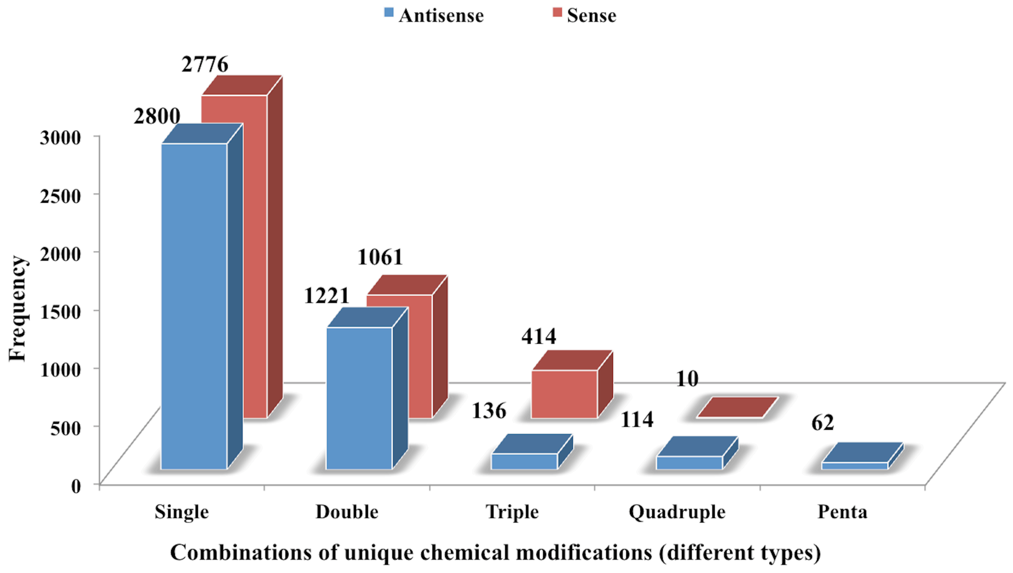

Figure S4. Grouping frequency of different unique chemical modifications i.e. how many different modifications are grouped in cm-siRNAs.

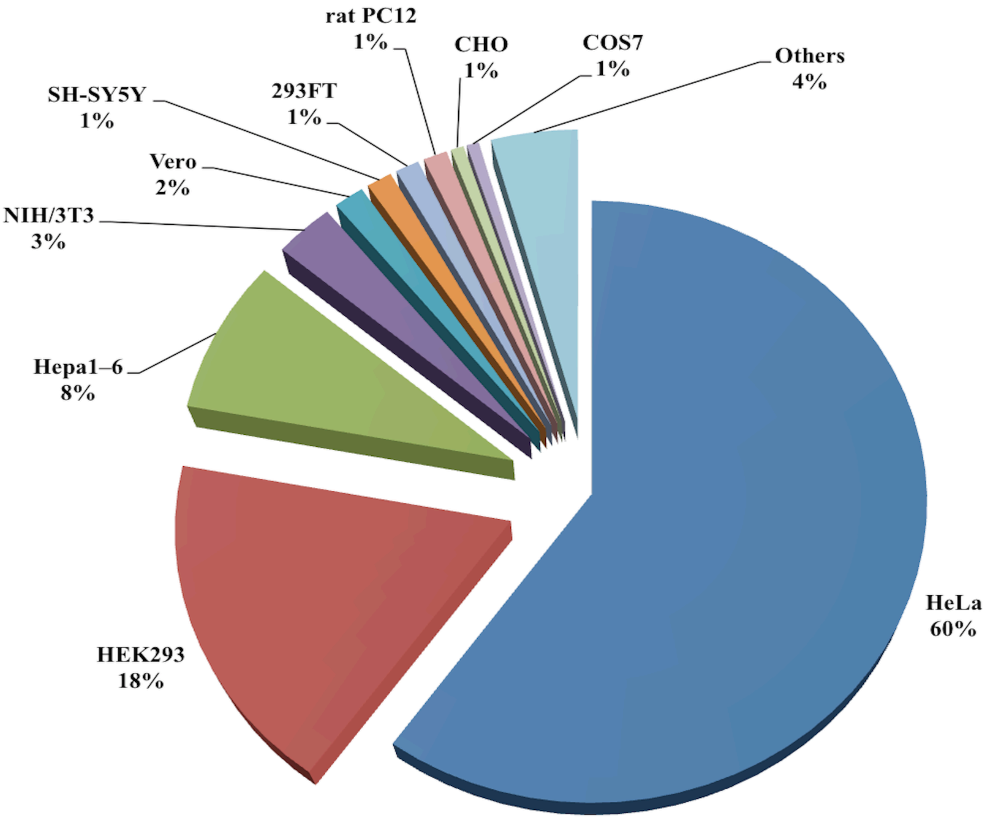

Figure S5. Cell lines distribution in siRNAmod database.
